# New Cell Lines Derived from Laboratory Colony *Triatoma infestans* and *Rhodnius prolixus*, Vectors of *Trypanosoma cruzi*, Do Not Harbour Triatoma Virus

**DOI:** 10.3390/insects13100906

**Published:** 2022-10-05

**Authors:** Rebekah Penrice-Randal, Catherine Hartley, Alexandra Beliavskaia, Xiaofeng Dong, Luke Brandner-Garrod, Miranda Whitten, Lesley Bell-Sakyi

**Affiliations:** 1 Department of Infection Biology and Microbiomes, Institute of Infection, Veterinary and Ecological Sciences, University of Liverpool, 146 Brownlow Hill, Liverpool L3 5RF, UK; 2 Department of Infection Biology, London School of Hygiene and Tropical Medicine, Keppel Street, London WC1E 7HT, UK; 3 Swansea University Institute of Life Science, College of Medicine, Swansea University, Singleton Park, Swansea SA2 8PP, UK

**Keywords:** *Triatoma infestans*, *Rhodnius prolixus*, insect, vector, kissing bug, cell line, Triatoma virus

## Abstract

**Simple Summary:**

Arthropod vector cell lines play an important role in research on the biology and control of blood-feeding insects and the diseases they transmit. Kissing bugs of the genera *Triatoma* and *Rhodnius* transmit *Trypanosoma cruzi*, the causative agent of Chagas disease in humans in tropical Latin America. We have developed new cell lines from two of the major vectors of *T. cruzi*, *Triatoma infestans* and *Rhodnius prolixus*, and have shown that they do not harbour any contaminating bacteria or known insect-borne viruses. We propose that the new cell lines may be suitable host cells for Triatoma virus, a natural pathogen of *T. infestans* that has been proposed as a biocontrol agent but cannot be grown in existing cell lines. The new cell lines may also be used to study interactions between trypanosomes and cells of their vectors.

**Abstract:**

Triatomine bugs of the genera *Triatoma* and *Rhodnius* are vectors of Chagas disease, a neglected tropical disease of humans in South America caused by *Trypanosoma cruzi*. Triatoma virus (TrV), a natural pathogen of *Triatoma infestans*, has been proposed as a possible tool for the bio-control of triatomine bugs, but research into this virus has been hampered by a lack of suitable host cells for *in vitro* propagation. Here we report establishment and partial characterisation of continuous cell lines from embryos of *T. infestans* (TIE/LULS54) and *Rhodnius prolixus* (RPE/LULS53 and RPE/LULS57). RNAseq screening by a sequence-independent, single primer amplification approach confirmed the absence of TrV and other RNA viruses known to infect *R. prolixus*, indicating that these new cell lines could be used for propagation of TrV.

## 1. Introduction

Triatomine bugs of the order Hemiptera, family Reduviidae, also known as kissing bugs, are vectors of the kinetoplastid protozoan parasite *Trypanosoma cruzi*. *T. cruzi* is the causative agent of Chagas disease, a zoonosis widespread in Latin America [1]. Following hatch of first-instar nymphs from eggs, all the developmental stages of triatomine bugs are blood-feeding, thereby causing irritation to their hosts as well as the risk of pathogen transmission. The control of triatomine bugs currently depends on insecticide treatment of infested dwellings; however, resistance to many commonly-used insecticides has been reported in multiple South American countries [2], indicating a need to explore other control strategies.

Triatoma virus (TrV) [3], a member of the family Dicistroviridae [4] in the order Picornavirales, has been proposed as a possible agent for the bio-control of triatomine bugs [5]. TrV causes a fatal disease in colony-reared *Triatoma infestans* [6] and there is some evidence that prior TrV infection of *T. infestans* increases their *T. cruzi* infection rate [7]. TrV infection was reported in 10% of field-collected *T. infestans* in Argentina [6] and in several laboratory colonies in Argentina and Brazil [6,8]. Interestingly, TrV was also detected in a laboratory colony of another triatomine bug, *Rhodnius prolixus* [8]. Research into TrV has been hampered by the lack of an *in vitro* system for laboratory propagation of the virus outside the insect host [9]. To date, no insect cell line has been reported to support replication of TrV [10], possibly because the virus may be specific to triatomine bugs [11], and no cell lines derived from *Triatoma* or *Rhodnius* spp. were available to test.

Primary *Triatoma maculatum* embryo-derived cell cultures surviving for up to 49 days were reported over 50 years ago [12], and a continuous cell line was subsequently generated from embryos of *T. infestans* [13]. This cell line, TI-32 (originally designated BTC-32) supported in vitro differentiation of *T. cruzi* [14] and continuous growth of the microsporidian fungus *Nosema disstria* [15]. However, the TI-32 cell line is not currently available from any culture collection and is no longer held at its place of origin, the London School of Hygiene and Tropical Medicine (LSHTM). No cell lines have been reported from any species of the genus *Rhodnius*.

Here we report the establishment and partial characterisation of three continuous cell lines derived from embryos of *T. infestans* and *R. prolixus* obtained from laboratory colonies. Screening by a sequence-independent, single primer amplification (SISPA) approach [16] revealed that none of the cell lines harbour TrV or other RNA viruses previously reported from *R. prolixus* [17].

## 2. Materials and Methods

### 2.1. Insects

*T. infestans* eggs were harvested from a laboratory colony maintained at LSHTM since 2009. The colony originated from *T. infestans* insects collected in Paraguay by Dr Nidia Acosta, Universidad Nacional de Asunción—UNA, Paraguay. The *T. infestans* insects were maintained in glass jars measuring 9.5 cm × 14 cm at 26 °C ± 2 °C, at 70% relative humidity with a 12 h light:12 h dark cycle, and fed every 6–8 weeks on defibrinated horse blood (TCS Microbiology, Buckingham, UK) using a Hemotek^®^ membrane feeding system with 5 mL reservoirs (Hemotek Ltd., Blackburn, UK). *R. prolixus* eggs were harvested from a laboratory colony maintained at Swansea University [18]. This colony was derived from a colony of insects originating from Venezuela and maintained at LSHTM since the late 1920s [19]. The *R. prolixus* insects were fed via a membrane feeding system on human blood. Whole blood was collected by phlebotomists at the School of Medicine, Swansea University, from anonymous healthy adult volunteers by venipuncture into vacuette^®^ tubes containing CPDA (Greiner Bio-One, Stonehouse, UK). All human blood sampling procedures were reviewed and approved by the South West Wales Research Ethics Committee, REC ref 13/WA/0910. For both insect species, the eggs were used to generate primary cell cultures when the eyes of the majority of developing embryos were visible as reddish spots inside the eggshells.

### 2.2. Preparation of Primary Cell Cultures

Three complete culture media were used: L-15 (Leibovitz) medium supplemented with 10% tryptose phosphate broth, 20% foetal bovine serum (FBS), 2 mM L-glutamine (L-glut), and 100 units/mL of penicillin and 100 µg/mL of streptomycin (antibiotics) (L-15); Hanks balanced salt solution (HBSS) supplemented with 0.5% lactalbumin hydrolysate, 20% FBS, L-glut and antibiotics (H-Lac); and a 1:1 mixture of L-15 and H-Lac (L-15/H-Lac). Complete media were prepared freshly each week. All ingredients were sourced from Invitrogen (Thermo-Fisher, Loughborough, UK) or Sigma-Aldrich (Gillingham, UK). 

*T. infestans* and *R. prolixus* eggs were surface-sterilised by immersion in 0.1% benzalkonium chloride for 1–3 min, 70% ethanol for 1 min, sterile ultrapure water for 10 s and HBSS for 10 s in a 30 mm plastic petri dish. The eggs were immersed in fresh HBSS and dissected aseptically by carefully removing the operculum of each egg and gently squeezing out the embryo using sterile watchmakers’ forceps under a binocular dissecting microscope in a horizontal laminar flow cabinet. Embryos with pale or red eye spots were selected for further processing; older embryos with black eye spots (both species) and/or developing reddish exoskeleton (*R. prolixus*) were rejected. The embryos were collected in a drop of HBSS in a second petri dish and macerated using the watchmakers’ forceps. The macerated tissues were diluted with 1 mL of HBSS and centrifuged at 200× *g* for 5 min. The tissue pellet was resuspended in 0.5 mL of a solution of 500 µg/mL trypsin (Sigma-Aldrich, Gillingham, UK) in PBS and incubated at 37 °C for 5 min (*R. prolixus*) or 10 min (*T. infestans*). Then 1 mL of complete culture medium was added to inactivate the trypsin and the tissue suspension was centrifuged as before. The tissue pellet was resuspended in 2.2 mL of complete culture medium and placed in a flat-sided culture tube (Nunc, Thermo-Fisher, Loughborough, UK). The sealed tubes were incubated in ambient air at 28 °C. Medium was changed weekly by removal and replacement of ¾ of the total volume. Cultures were monitored for growth by weekly inverted microscope examination. 

### 2.3. Subculture and Cell Line Generation

When significant cell multiplication became evident, subcultures were initiated. An equal volume of fresh complete medium (2.2–2.4 mL) was added to the culture, adherent cells and tissue clumps were resuspended by pipetting and half of the resultant suspension was transferred to a new daughter tube, leaving the remainder in the parent tube. When sufficient numbers of tubes were available, half of the contents of two tubes were pooled and transferred to a T25 flask with a total of 5.5 mL of medium. The subculture series were maintained thereafter in parallel in tubes and T25 flasks; cells in flask cultures were passaged 1:1 by resuspending the monolayer using a cell scraper instead of pipetting. Occasionally, cells were resuspended and used to prepare Giemsa-stained cytocentrifuge smears as described previously [20].

### 2.4. Cryopreservation and Resuscitation of Cultured Cells

Cultured cells were cryopreserved using a modification of a previously-described technique [21], as follows. Cells were harvested from tubes by pipetting or from flasks by scraping and pooled and centrifuged at 300× *g* for 5 min. The cell pellet was resuspended in ice-cold complete medium, held on ice and an equal volume of ice-cold complete medium with 20% DMSO was added dropwise with swirling. The resultant suspension was immediately dispensed as 1 mL aliquots into ice-cold cryovials and frozen rapidly on dry ice, before being transferred to vapour-phase liquid nitrogen storage within 30 min. The contents of three culture tubes or one T25 flask yielded three cryovials. Cells were resuscitated from liquid nitrogen storage by rapid thawing in a 37 °C water bath, added immediately to 9 mL of complete medium at room temperature, centrifuged at 300× *g* for 5 min, resuspended in 2.2 mL of complete medium, placed in a flat-sided tube and incubated at 28 °C. The first medium change was carried out after 5–7 days.

### 2.5. Species Confirmation and Screening for Contaminating Bacteria

Genomic DNA was extracted from harvested cells using a DNeasy Blood and Tissue Kit (Qiagen, Hilden, Germany) following the manufacturer’s instructions for tissue cultures and stored at −20 °C. To confirm the species origin, a fragment of the eukaryotic 18S rRNA gene was amplified by a conventional PCR from the *T. infestans* and *R. prolixus* cell DNA using forward primer EukA: 5′-AACCTGGTTGATCCTGCCAGT-3′ and reverse primer EukB: 5′-TGATCCTTCTGCAGGTTCACCTAC-3′ [22]. PCR reactions were carried out in a volume of 50 µL containing 25 ng of template DNA, 25 µL of Q5 High-Fidelity 2X Master Mix (New England Biolabs, Hitchin, UK), 5 µL of each primer at 10 mM concentration and clean water to make up the final reaction volume. The thermal cycling conditions were: 96 °C for 5 min, 30 cycles of 96 °C for 15 s, 60 °C for 30 s, 72 °C for 2 min and a final elongation at 72 °C for 5 min. Additional PCRs targeting triatomine ITS-2 [23] and *cytB* [24] genes were carried out to refine the species identification, following the published protocols. To detect any bacterial contaminants, a conventional pan-bacterial PCR assay targeting a ~1500 bp fragment of the 16S rRNA gene with the primer pair fD1 and rP2 was carried out as described previously [25]. All PCR assays included negative controls (water instead of test DNA) and the pan-bacterial 16S rRNA PCR included positive control DNA extracted from an *Ehrlichia ruminantium*-infected bovine endothelial cell culture [26]. PCR products were detected by agarose gel electrophoresis; positive amplicons were purified using a PureLink Quick Gel Extraction and PCR Purification Combo kit (Thermo-Fisher, Loughborough, UK) following the manufacturer’s instructions and submitted to Sanger sequencing in both directions (Eurofins Genomics, Ebersberg, Germany). The sequences were analysed in Bioedit v.7.2.5 [27]. Randomly-selected *T. infestans* and *R. prolixus* cultures were screened for contamination with *Mycoplasma* spp. using two commercial tests, the Mycoalert Mycoplasma Detection Kit (Lonza, Thermo-Fisher, Loughborough, UK) and the PCR Mycoplasma Test Kit (Promocell, VWR, Lutterworth, UK), following the manufacturers’ instructions.

### 2.6. SISPA Screening for TrV and Other RNA Viruses

Total RNA was extracted from cells harvested from two flat-sided tubes or one T25 flask using an RNeasy blood and tissue kit (Qiagen, Hilden, Germany) following the manufacturer’s instructions. Following a TurboDNase treatment, the RNA was reverse transcribed using SuperScript IV Reverse Transcriptase (Invitrogen, Thermo-Fisher, Loughborough, UK) using primer Sol-A, according to the manufacturer’s instructions. Klenow Fragment (New England Biolabs, Hitchin, UK) was used for second strand synthesis [16]. The cDNA was amplified with Q5 High-Fidelity DNA Polymerase (New England Biolabs, Hitchin, UK) with Primer Sol-B. The cycling conditions were as follows; 98 °C for 30 s, followed by 30 cycles of 98 °C for 10 s, 55 °C for 30 s and 72 °C for 1 min, with a final extension at 72 °C for 10 min. PCR products were purified using AMPure XP beads and then quantified using the 1X dsDNA HS kit with the Qubit fluorometer. Then, Nanopore sequencing libraries were prepared as described in the SQK-LSK109 protocol with native barcoding EXP-NBD104. The libraries were loaded onto R.9.4.1. flow cells and a sequencing run was first carried out for 6 h 19 min; a second run of each sample was carried out for 46 h to obtain a greater sequence depth. The GridION basecalled the sequencing reads in real-time using the high-accuracy parameters. A negative control consisting of nuclease-free water (Thermo-Fisher, Loughborough, UK) only was included to identify environmental contamination and sequencing cross-talk.

NanoFilt was used to filter out sequencing readings less than 100 nucleotides and to then trim 30 nucleotides off the end of the read using the --headcrop and --tailcrop parameters [28]. The first run of each sample was analysed with gb_taxonomy_tools https://github.com/spond/gb_taxonomy_tools (accessed on 29 January 2022) based on taxids searched by BLAST and visualised with Krona [29]. For the second runs, filtered FASTQ files were then uploaded to the Bugseq platform https://bugseq.com/free (accessed on 29 January 2022) using the NCBI nt database and visualised with Recentrifuge [30].

### 2.7. Deposition of Sequences in Public Databases

Sequences obtained from PCR products sequenced successfully in both directions were deposited in GenBank with accession numbers OP123038 (TIE/LULS54) and OP123039 (RPE/LULS53) for the eukaryotic 18S rRNA gene. The SISPA RNAseq datasets obtained for the three cell lines were deposited in the NCBI Sequence Read Archive (SRA) database under BioProject number PRJNA843092.

## 3. Results

### 3.1. T. infestans Cell Line TIE/LULS54

Three *T. infestans* primary cultures, one in H-Lac and two in L-15 derived from a single batch of ~90 eggs, were set up in flat-sided tubes and survive at the time of writing (30 months post-initiation). The primary cultures comprised adherent tissue clumps, some clearly identifiable as sections of limbs, and individual cells with a range of phenotypes including round, epithelial-like, fibroblast-like and contracting muscle cells. As time passed, epithelial-like and muscle cells came to predominate in all cultures. Subculture series were initiated from two of the primary cultures, the one in H-Lac and one of the ones in L-15, after 12 and 9 weeks respectively; growth of the H-Lac culture was slow and this series only reached passage 3 after a total of two years in culture. 

The L-15 culture grew more rapidly, reaching passage 2 after a further 6 weeks and giving rise to a continuous cell line, designated TIE/LULS54. At passage 3, cells of this line were transferred to a T25 flask, where they grew almost as well as in the flat-sided tubes and were easier to dislodge for subculture using a cell scraper. At 10 months after initiation, cells at passage 3 were cryopreserved and successfully resuscitated, and DNA was extracted from a passage 1 culture at that time and from a passage 8 culture at 22 months, for species confirmation and microorganism screening. At 20 months after initiation, RNA was extracted from two passage 6 cultures for RNAseq analysis. At the time of writing, the TIE/LULS54 cell line has reached passage 14; subcultures are carried out at 6-week intervals.

**Figure 1:**
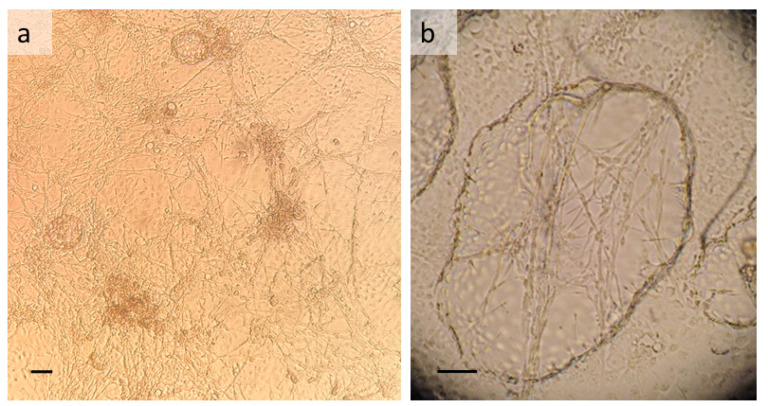
*Triatoma infestans* embryo-derived cell line TIE/LULS54. (**a**) General view of monolayer at passage 6; (**b**) blister-like multicellular dome formed above the cell monolayer in a ten-week-old passage 3 culture. Live, inverted microscope; scale bars 100 µm.

TIE/LULS54 cultures comprise a mixture of phenotypes (Figure 1a), including sheets of epithelial-like cells, actively-contracting muscle cells arranged in a lattice, and large rounded cells. Following subculture, the cells take 4–6 weeks to achieve confluency, but already within the first week the muscle cells have resumed their contractions. Once the cells are confluent, blister- or dome-like structures formed from sheets of epithelial-like cells arise from the cell monolayer (Figure 1b). These normally remain attached to the basal monolayer, only very rarely detaching to form floating multicellular vesicles. The muscle cells appear to contract continuously, only becoming quiescent immediately after a medium change or sometimes when exposed to bright light (as when examined by inverted microscope). TIE/LULS54 cultures can be maintained with weekly medium change but without subculture for at least 18 months, during which time the structures described above become increasingly complex and muscle contractions are maintained in parts of the culture. The movements of the lattice of contracting muscle cells (Video S1) may be so large as to be visible to the naked eye.

### 3.2. R. prolixus Cell Lines RPE/LULS53 and RPE/LULS57

Eleven *R. prolixus* primary cultures were initiated from batches of 12–80 eggs; of these, two in L-15/H-Lac and two in L-15 survive at the time of writing (38–40 months after initiation). In the failed primary cultures, the cells and tissue clumps did not attach well to the surface of the culture tube and did not metabolise beyond the first few weeks, as indicated by rising pH of the culture medium. In the successful primary cultures, tissue clumps attached within the first few days and cells began to migrate out from them, forming sheets of epithelial-like cells and small patches of contracting muscle cells. Areas of large epithelial-like cells containing red pigment, visible to the naked eye, were present in some of the cultures; these persisted for more than 20 months in one of the surviving L-15 cultures. In contrast, the areas of muscle cells ceased to contract after 12–18 months. Attached, and occasionally floating, multicellular vesicles appeared in most of the primary cultures after several months, but did not persist to the time of writing. The subculture series were initiated from three of the surviving primary cultures after 10, 13 and 26 months; of these, the first two gave rise to the continuous cell lines described below while the third, in L-15, has reached passage 4.

The first of the two *R. prolixus* cell lines to become established, RPE/LULS53, was generated from 30 eggs in L-15. The first subculture was carried out at 10 months after initiation, with the second and third passages following at 5- and 3-month intervals respectively. Cells at passage 4 were transferred to a T25 flask to facilitate subculturing, 19 months after initiation. The first cryopreservation attempt, carried out with passage 4 cells 20 months after initiation, was successful. DNA was extracted from a passage 1 culture at that time, and from a passage 13 culture at 33 months, for species confirmation and microorganism screening; at 32 months after initiation, RNA was extracted from a passage 12 culture for RNAseq analysis. The RPE/LULS53 cells have reached passage 20 at the time of writing.

The second *R. prolixus* cell line, designated RPE/LULS57, was generated from 44 eggs in L-15/H-Lac. The first subculture was carried out after 19 months, passage 3 cells were successfully cryopreserved at 23 months and the cells have reached passage 14 at the time of writing. DNA was extracted from a passage 6 culture at 30 months for species confirmation and microorganism screening, and RNA for RNAseq was extracted at 29 months from pooled cultures at passages 5 and 7.

**Figure 2:**
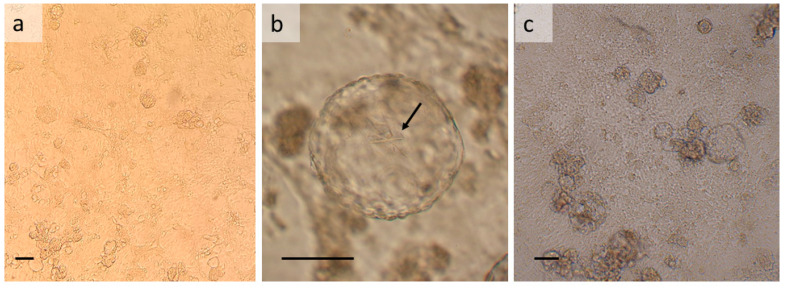
*Rhodnius prolixus* embryo-derived cell lines RPE/LULS53 and RPE/LULS57. (**a**) General view of RPE/LULS53 at passage 8; (**b**) needle-shaped and rectangular crystals inside a RPE/LULS53 multicellular vesicle at passage 15; (**c**) general view of RPE/LULS57 cells at passage 12. Live, inverted microscope; scale bars 100 µm.

Both *R. prolixus* cell lines comprise cells that are considerably smaller than those of the *T. infestans* line TIE/LULS54 and do not exhibit muscle contractions. Several cell types are present, forming a monolayer of spindle-shaped and fibroblast-like cells (Figure 2a,c). Multicellular vesicles start to form within a week of the subculture; these remain firmly attached to the monolayer and in older (>4 weeks) RPE/LULS53 cultures, needle-shaped crystals of unknown composition form inside some of the vesicles (Figure 2b). In RPE/LULS57 cultures, attached clusters of multiple smaller vesicles are also present. Free-floating multicellular vesicles are rarely seen. The *R. prolixus* cell lines can be subcultured by pipetting or scraping at 2-week intervals. 

### 3.3. Confirmation of Species Origin and Screening for Contaminating Microorganisms

For the TIE/LULS54 cells, the amplicons obtained using the PCR targeting the eukaryotic 18S rRNA gene yielded a 1808 bp sequence with 99.83–99.89% similarity to sequences deposited in GenBank from *Triatoma sordida* and *T. infestans* (Table 1). The 18S rRNA sequence obtained from the RPE/LULS53 cells spanned 1775 bp and showed 99.83–99.94% identity (100% query cover) to those deposited in GenBank from *Rhodnius stali* and *R. prolixus* (Table 1). In both cases, the closest match was not the expected species, so PCRs targeting two additional triatomine genes, ITS-2 and *cytB*, were carried out on all three cell lines. For both genes and all three cell lines, the top BLAST hits were sequences from the expected species (Table 2).

The pan-bacterial 16S rRNA PCR failed to amplify any products from TIE/LULS54, RPE/LULS53 or RPE/LULS57, while a product of the expected size was amplified from the positive control *E. ruminantium* DNA. All cell lines were negative for *Mycoplasma* spp. in both of the commercial tests.

The Nanopore sequencing yielded between 0.1 and 0.6 million reads in the first run and 3.5 and 4.1 million reads in the second run for each cell line, with mean read lengths between 752 and 940 bases (Table 2). The proportions of reads assigned to viruses was very low for all three cell lines, ranging from 0.0001% to 0.02%. An analysis of the reads by both approaches did not yield any sequences that matched with the 864 TrV sequences available in GenBank at the time of writing, indicating that TrV was not present in any of the three cell lines. Moreover, none of the reads matched with any of the seven novel RNA viruses reported previously from *R. prolixus* [17].

## 4. Discussion

In the present study, we generated continuous cell lines from two medically-important triatomine bug species. The approach used to generate the TIE/LULS43, RPE/LULS53 and RPE/LULS57 cell lines was relatively simple, and based on techniques used previously to generate a range of insect and tick cell lines [13,21,37,38]. No attempt was made to select specific insect organs as a starting material, and the resultant cell lines are phenotypically heterogeneous, all clearly comprising multiple cell types. An interesting characteristic of TIE/LULS54 is the continuous presence over 2.5 years and 14 passages of contractile cells, recently highlighted as being of rare occurrence among insect cell lines [39]. It remains to be seen if this characteristic will be retained in higher passage levels. While the *T. infestans* cells became established in a shorter time than the *R. prolixus* cells (10 months compared to 19–23 months), the *R. prolixus* cell lines now grow faster than the *T. infestans* cell line. Further study would be required to investigate these contrasting characteristics. All three cell lines are grown in relatively simple culture media, for which all ingredients are commercially available, and are incubated in sealed containers in ambient air, making them suitable for use in laboratories with only basic cell culture facilities and therefore making them applicable in a range of settings in countries where *T. cruzi* and its vectors are endemic.

*T. infestans* and *R. prolixus* are among the four main vectors of *T. cruzi* in Latin America [40] and, as such, their control is a major part of the overall strategy to minimise or prevent the occurrence of Chagas disease in the human population of endemic areas. The availability of triatomine cell lines will contribute to a better understanding of the relationship between the trypanosomes and their vectors and potentially to the development of improved insect control methods. In the only previous study on co-cultivation of *T. cruzi* with triatomine cells, trypanosomes cultured with *T. infestans* TI-32 cells underwent a pattern of differentiation similar to that seen in the vector but not seen in axenically-cultured trypanosomes, indicating that the TI-32 cells produced factor(s) influencing parasite development [14]. TI-32 cells were also applied to the isolation and study of a strain of the reptilian parasite *Trypanosoma grayi*, isolated from a *Glossina pallipides* tsetse fly, that could not be maintained in axenic culture [41,42]. The infectivity of four strains of *T. cruzi* for mammalian and insect cells was compared using the non-vector *Aedes albopictus* mosquito cell line C6/36; in all cases the percentage of cells infected was lower in C6/36 cells than in mammalian cells, but the numbers of parasites associated with the infected cells was similar overall [43]. It will be interesting to characterise the interaction between *T. cruzi* and the new triatomine cell lines TIE/LULS54, RPE/LULS53 and RPE/LULS56.

Triatomine bugs also harbour trypanosome species that are not known to infect humans but may be pathogenic for their insect hosts and potentially modulate infection with *T. cruzi* [18,44]. TI-32 cells were found to be essential for isolation and propagation of *Blastocrithidia triatomae*, originally isolated from *T. infestans* in Argentina; interaction with the trypanosomes appeared to kill the TI-32 cells [44]. Studies on interaction between two strains of *Trypanosoma rangeli* and *R. prolixus* haemocytes in vivo and ex vivo [18] suggest that *R. prolixus* cell lines could be exploited to further examine the role of reactive oxygen species in the pathogenicity of *T. rangeli* for its host. In addition to trypanosomes, triatomine bugs could play a role in the transmission of bacterial pathogens such as *Bartonella* spp. and *Mycobacterium leprae*, the causative agent of leprosy [45,46]. Indeed, the slow growth rate and ability to survive without a regular subculture suggest that the new *T. infestans* and *R. prolixus* cell lines might prove to be suitable host cells for the *in vitro* propagation of *M. leprae*, as reported previously for a tick cell line [47]. *T. infestans* may also harbour symbiotic intracellular bacteria of the genus *Arsenophonus*; *Candidatus* Arsenophonus triatominarum was isolated from adult female *T. infestans* haemolymph and propagated in a mosquito cell line [48]. As the TIE/LULS54 cell line is free of *Ca.* A. triatominarum, these cells could be used as a more biologically-relevant substrate than mosquito cells for in vitro propagation and the study of this symbiont.

The environmental costs of insecticides and the increase in reports of insecticide resistance among triatomine bug populations across South America [2,49,50] underline the need for new approaches to vector control. Bio-control, exploiting natural pathogens of triatomines such as TrV as control tools, is an attractive approach; however, the experience with the fungal pathogen *Metarhizium anisopliae*, which took longer to kill bugs infected with *T. cruzi* than uninfected bugs [51] indicates the need for caution. Similarly, the reported increase in the susceptibility to *T. cruzi* infection in TrV-infected *T. infestans* [7] deserves further investigation to determine the effect of co-infection on transmission of the parasite to a vertebrate host.

The reported failure to propagate TrV in cell lines derived from non-triatomine insects [10], and the failure to infect mosquitoes, honey bees and mice with TrV [11,52] underscore the need for an in vitro culture system for TrV that would allow large-scale laboratory production of the virus to support research into its potential as a bio-control tool. While it is not yet known whether or not any of the new *T. infestans* and *R. prolixus* cell lines will support productive infection with TrV, we have carried out the first step in this journey by screening them for an existing TrV infection.

The Nanopore sequencing platform was applied in preference to the RT-PCR assays used by others to screen laboratory and field populations of *T. infestans* and *R. prolixus* for TrV [5,8], as we did not have access to a positive control sample. Following an initial run in which we obtained low proportions and actual numbers of virus sequences, we carried out a second, longer run on all the samples to obtain greater depth in our analysis. Analysis of this second run produced even lower proportions of virus sequences. Our approach not only confirmed the absence of TrV but also confirmed the absence of RNA viruses of the families Iflaviridae, Solemoviridae and Permutotetraviridae, previously reported to be harboured by *R. prolixus* [17]. Interestingly, the proportion of reads identified as putative viral sequences by both bioinformatics pipelines in all three cell lines (0.0001–0.02%) was much lower than that reported previously [17] in RNA extracted from whole *R. prolixus* insects (0.1–0.6%). These authors used a different approach, generation of paired-end Illumina RNAseq libraries and Illumina HiSeq sequencing, which could have influenced the proportions of viral reads detected in their insect samples. Of likely greater significance, however, are the different origins of the insects used in our study and that of Brito and co-workers [17]. Their *R. prolixus* came from a laboratory colony which originated in Brazil, presumably from insects collected from the field in Brazil, sometime prior to 1980. Our *R. prolixus* came from a laboratory colony originated with insects from Venezuela nearly 100 years ago [19,53]; it is therefore unlikely that they would have harboured a virome identical to insects from Brazil at the time of the colony initiation, and it is possible that their virome became depleted over the many generations in the laboratory. Moreover, the effect on any existing virome of maintenance as primary cultures and cell lines is unknown.

The *T. infestans* cell line TIE/LULS54 harboured the least diverse virome of the three cell lines, as determined by RNAseq, with between 0.0001 and 0.0005% of reads assigned to putative viral sequences, indicating the absence of any complete, replication-competent RNA viruses. This finding suggests that TIE/LULS54 cells would be particularly useful not only for the propagation of TrV, but also for more general investigation of insect host cell responses to RNA virus infection, as any response detected following infection of the cells could be confidently attributed to the presence of the test virus.

In conclusion, the three new triatomine cell lines reported here represent research tools with a high potential for application in studies both on bio-control using TrV and on interaction of *T. cruzi* and other trypanosomes with their vectors at the cellular level. All three cell lines are deposited in, and available from, the Tick Cell Biobank at the University of Liverpool.

## Figures and Tables

**Table 1:** Confirmation of species origin of *Triatoma infestans* cell line TIE/LULS54 and *Rhodnius prolixus* cell lines RPE/LULS53 and RPE/LULS57. Closest identity with published sequences in GenBank obtained by BLAST analysis of sequences amplified using PCRs targeting Eukaryotic 18S (Euk 18S) and triatomine rDNA second internal transcribed spacer (ITS-2) and cytochrome B (*cytB*) genes.

Cell Line	Gene Targeted	Sequence Length	Top BLAST Hits (GenBank Accession Number)	Query Cover	% Identity (Identical/Total bp)	Reference
TIE/LULS54	Euk 18S	1807 bp	*Triatoma sordida*, Bolivia (AJ421956)	100%	99.89% (1807/1809)	[31]
			*T. infestans*^b^ (Y18750)		99.83% (1807/1810)	[32]
	ITS-2 ^a^	533 bp	*T. infestans*, Brazil (AY860388)	87%	98.71% (460/466)	[33]
	*cytB* ^a^	666 bp	*T. infestans*, Uruguay (KY640305)	52%	84.73% (294/347)	[34]
			*T. infestans* Paraguay (KY654076)		84.44% (293/347)	[35]
RPE/LULS53	Euk18S	1775 bp	*Rhodnius stali*, Bolivia (AJ243335)	100%	99.94% (1774/1775)	[32]
			*R. prolixus*, Brazil (AJ421962)		99.83% (1772/1775)	[31]
	ITS-2 ^a^	420 bp	*R. prolixus*, Colombia (AY345868)	50%	81.52% (172/211)	Unpublished ^c^
	*cytB* ^a^	661 bp	*R. prolixus*, Colombia (MH704763)	36%	89.92% (214/238)	[36]
RPE/LULS57	ITS-2 ^a^	646 bp	*R. prolixus*, Colombia (AY345868)	49%	67.09% (210/313)	Unpublished ^c^
	*cytB* ^a^	189 bp	*R. prolixus*, Colombia (MH704763)	97%	94.02% (173/184)	[36]

^a^ Analysis based on sequence obtained with one primer only; sequence available upon request. ^b^ Unspecified geographic origin. ^c^ Deposited in GenBank by Vergel MC, Jaramillo CA, Vallejo GA and Guhl F.

**Table 2:** Summary of RNAseq data obtained by Nanopore sequencing of RNA extracted from *Triatoma infestans* cell line TIE/LULS54 and *Rhodnius prolixus* cell lines RPE/LULS53 and RPE/LULS57. Two sequencing runs were carried out.

Cell Line Sequencing Data	TIE/LULS54	RPE/LULS53	RPE/LULS57	Negative Control
Number of reads in first run	630037	453711	103105	4386
Number of reads in second run	4124079	3874280	3464787	54662
Mean read length	751.9	782.4	940.3	802
Mean read quality	13.4	13.4	13.4	13.3
Number of reads assigned to viruses in first run	3 (0.0005%)	16 (0.004%)	17 (0.02%)	10 (0.2%)
Number of reads assigned to viruses in second run	4 (0.0001%)	5 (0.0001%)	18 (0.0005%)	39 (0.07%)

## Data Availability

Sequences obtained from PCR products sequenced successfully in both directions were deposited in GenBank with accession numbers OP123038 (TIE/LULS54) and OP123039 (RPE/LULS53) for the eukaryotic 18S rRNA gene. The SISPA RNAseq datasets obtained for the three cell lines were deposited in the NCBI Sequence Read Archive (SRA) database under BioProject number PRJNA843092. Sequences obtained from PCR products that could only be sequenced in one direction are available from the authors on request.

## Supplementary Materials

The following supporting information can be downloaded at: https://www.mdpi.com/article/10.3390/insects13100906/s1, Video S1: a one-year-old culture of the *Triatoma infestans* cell line TIE/LULS54 at passage 3, showing a continuously contracting lattice of muscle cells above a discontinuous monolayer of rounded cells.
