# Supplementary material for: New Cell Lines Derived from Laboratory Colony Triatoma infestans and Rhodnius prolixus, Vectors of Trypanosoma cruzi, Do Not Harbour Triatoma Virus
Source: Insects. 2022 Oct 5;13(10):906. doi: 10.3390/insects13100906 (PMC9603895; doi:10.3390/insects13100906)
Supplement: Supplementary file 1 [file insects-13-00906-s001.zip › insects-1929457-supplementary.pptx]

## Slide 1
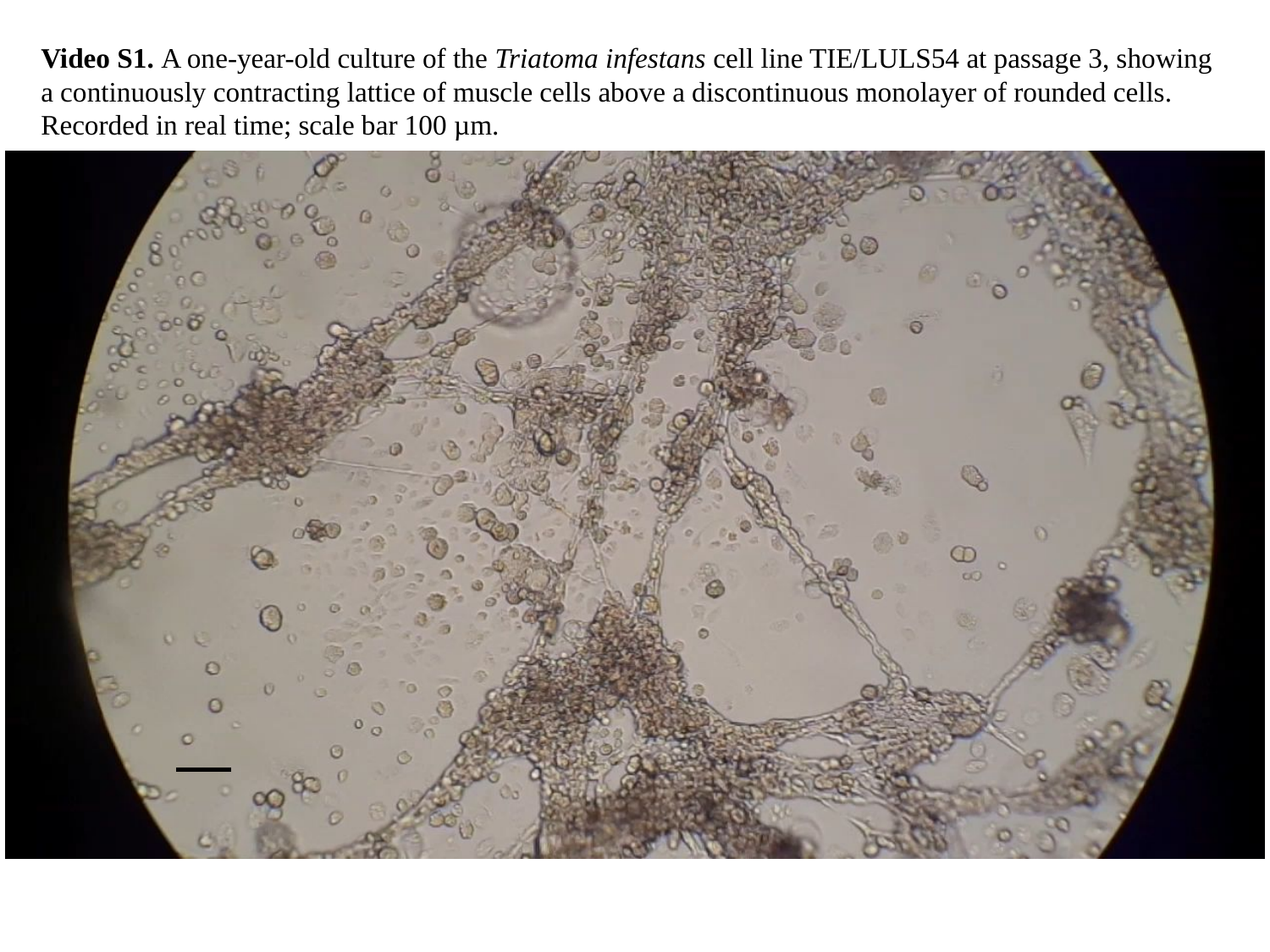

Video S1. A one-year-old culture of the Triatoma infestans cell line TIE/LULS54 at passage 3, showing a continuously contracting lattice of muscle cells above a discontinuous monolayer of rounded cells. Recorded in real time; scale bar 100 µm.
